# Automated Cancer Diagnostics via Analysis of Optical and Chemical Images by Deep and Shallow Learning

**DOI:** 10.3390/metabo12050455

**Published:** 2022-05-18

**Authors:** Olof Gerdur Isberg, Valentina Giunchiglia, James S. McKenzie, Zoltan Takats, Jon Gunnlaugur Jonasson, Sigridur Klara Bodvarsdottir, Margret Thorsteinsdottir, Yuchen Xiang

**Affiliations:** 1 Department of Metabolism, Digestion and Reproduction, Faculty of Medicine, Imperial College London, London SW7 2AZ, UK; ogi1@hi.is (O.G.I.); v.giunchiglia20@imperial.ac.uk (V.G.); j.mckenzie@imperial.ac.uk (J.S.M.); z.takats@imperial.ac.uk (Z.T.); 2 Faculty of Pharmaceutical Sciences, University of Iceland, Hofsvallagata 53, 107 Reykjavik, Iceland; 3 Biomedical Center, School of Health Sciences, University of Iceland, 101 Reykjavik, Iceland; skb@hi.is; 4 Department of Pathology, Landspitali the National University Hospital, Hringbraut, 101 Reykjavik, Iceland; jongj@landspitali.is; 5 Faculty of Medicine, University of Iceland, Vatnsmyrarvegur 16, 101 Reykjavik, Iceland

**Keywords:** mass spectrometry imaging, DESI-MSI, deep learning, shallow learning, FFPE, diagnostics

## Abstract

Optical microscopy has long been the gold standard to analyse tissue samples for the diagnostics of various diseases, such as cancer. The current diagnostic workflow is time-consuming and labour-intensive, and manual annotation by a qualified pathologist is needed. With the ever-increasing number of tissue blocks and the complexity of molecular diagnostics, new approaches have been developed as complimentary or alternative solutions for the current workflow, such as digital pathology and mass spectrometry imaging (MSI). This study compares the performance of a digital pathology workflow using deep learning for tissue recognition and an MSI approach utilising shallow learning to annotate formalin-fixed and paraffin-embedded (FFPE) breast cancer tissue microarrays (TMAs). Results show that both deep learning algorithms based on conventional optical images and MSI-based shallow learning can provide automated diagnostics with F1-scores higher than 90%, with the latter intrinsically built on biochemical information that can be used for further analysis.

## 1. Introduction

Universally, histology has been used to diagnose any disease involving changes in tissue structure. The analysis is based on a histopathologist´s observation of tissue morphology following staining [1]. The default workflow for histopathological analysis comprises formalin fixation and paraffin embedding (FFPE), as this treatment has been shown to preserve the tissue structure for many years [2,3]. The indefinite storage of FFPE samples while retaining its corresponding clinicopathological information makes these samples valuable and essential for clinical research [4,5]. From merely using haematoxylin and eosin (H&E) staining and periodic acid-Schiff staining to diagnose cancer, the typical workflow has become more complex, encompassing techniques such as immunohistochemistry (IHC) and molecular genetics [6]. Although scientists can recognise histological subtypes, only a qualified pathologist can correctly interpret and integrate biological, clinical, and morphological patterns of a studied disease. Over the last decade, the number of tissue blocks per case and the number of required slides per tissue block have increased by more than 60%, reflecting the ever increasing complexity of histopathology diagnostics [6,7]. This diagnostic workflow is time-consuming, costly, and susceptible to error due to a fundamental subjectivity that is observer-dependent, thus leading to a sensitivity of only around 70% [8]. As a result, there is a growing demand in cancer diagnosis for streamlined histopathology procedures driven by tissue biology, which existing standard histology platforms cannot meet [6,9,10].

As a potential solution for the observer subjectivity problem while interpreting morphological patterns, computational pathology has significantly progressed over the last few years, digitising the workflow for histopathologists to aid decision support and easing the annotation process [11]. Digitisation of the workflow including the optical imaging of slides as whole-slide images (WSIs) has facilitated computer-assisted diagnostics (CAD) utilising deep learning (DL) methodologies. These workflows are envisioned to improve the efficiency and accuracy of pathology services and, ultimately, to provide improved patient care [11]. While traditional histopathology requires the annotation of specific regions by visual inspection of individual images, commercially available digital pathology systems (whilst not approved for diagnostic use) automate this process by using machine learning methods for annotation. These methods are trained using a large number of visually annotated sections as training sets; hence, classification is based on the knowledge of hundreds or thousands of histopathology professionals. Examples of such software include Indica Lab’s Halo AI [12] and Visiopharm’s Ontotopix [13]. The general requirement regarding comprehensive annotations is certainly a drawback of the approach, especially given the subjective nature of histopathological assessment [14], with one alternative approach being the application of weakly supervised DL methods [11]. Weak supervision requires only slide-level annotation (e.g., does this slide contain any cancer cells?) but can still be used to provide comprehensive annotations of WSIs once properly trained. This approach allows a routine histopathology process to be significantly accelerated while its diagnostic accuracy is simultaneously improved.

In this vein, other alternative approaches have also been developed for histological assessment. One of these methods is mass spectrometry imaging (MSI) which has become a promising approach for histological diagnostics. MSI enables the spatially resolved chemical profiling of tissue sections, allowing for the identification and mapping of a wide range of biomolecules such as metabolites, lipids, peptides, and drugs. Since the introduction of MSI in the early 1960s, a wide range of MSI techniques have been developed and demonstrated to have high potential for biomedical research, as it allows both targeted and untargeted analysis to discover biomarkers [15,16]. Among these techniques is desorption electrospray ionisation mass spectrometry imaging (DESI-MSI), which is particularly suited to investigate the spatial distribution of metabolites due to the lack of tissue modification prior to analysis [17,18]. One of the advantages of DESI-MSI compared to other common MSI techniques is that it can be used under ambient conditions with minimal sample preparation, making it well-suited for the automated, direct tissue analysis [17,19,20]. Furthermore, DESI-MSI has been proven to be reproducible and repeatable for various sample types across multiple laboratories [21], which is a key factor for the method to be applicable for clinical research that involves a large amount of slides that will inevitably have to be imaged under contrasting conditions at different time points [19,22,23,24,25,26,27].

In this study, with the aim of streamlined histopathology, two promising approaches for automated cancer diagnostics were investigated. The first approach is in line with the recent trend of digital pathology, where DL was applied to optical images of FFPE breast tissue microarrays (TMAs). In contrast, the second approach utilises shallow learning to analyse DESI-MSI images of the same TMAs. The performance of both approaches is discussed and compared.

In Section 2, the results and a discussion of the deep learning and shallow learning approaches for the diagnostic of FFPE breast cancer tissue microarrays are presented. Using deep learning algorithms, we show that it is possible to differentiate cancerous breast tissue and normal breast tissue, which is in line with previously published artificial intelligence approaches for histopathology problems. Using DESI-MSI with shallow learning performs better than the DL and provides chemical information that can be used for more detailed analysis. In Section 3, the experimental and data analysis methods used are briefly introduced. Section 4 concludes the findings of this paper.

## 2. Results & Discussion

### 2.1. Optical Imaging-Based Deep Learning Approach

**Figure 1:**
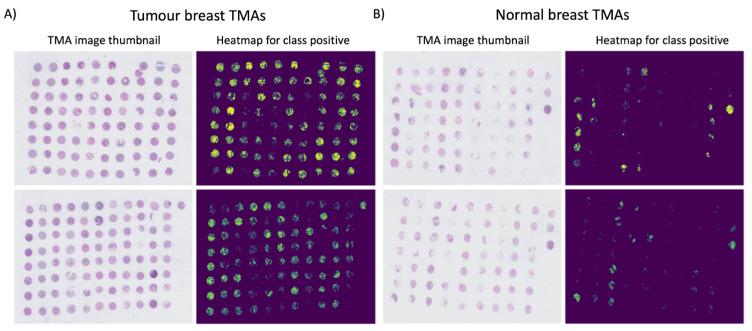
Probability heatmaps of tumour slides (**A**) and normal slides (**B**). The output probabilities for class positive predicted by the trained model on the TMA test set were used to reconstruct a heatmap of the full TMA slide. Each tile was represented as a 224 × 224 region within the image, which was then scaled to a thumbnail dimension. The heatmaps in (**A**,**B**) display only pixels with probabilities *p* > 0.5 for cancer prediction.

The DL classification algorithm was used to predict the probability of a 224 × 224 pixel tile being cancerous. Using these probabilities, a thumbnail-sized image was generated for each TMA and examples are displayed in Figure 1. As shown in the images, the model tends to classify most of the regions within tumour cores as being tumourous. In the case of normal slides, few regions within the normal cores are marked as tumourous. However, the areas marked as tumourous are much sparser and smaller in size compared to tumour slides and cores. These results suggest that the model might have a small bias towards the prediction of tumour cores.

**Figure 2:**
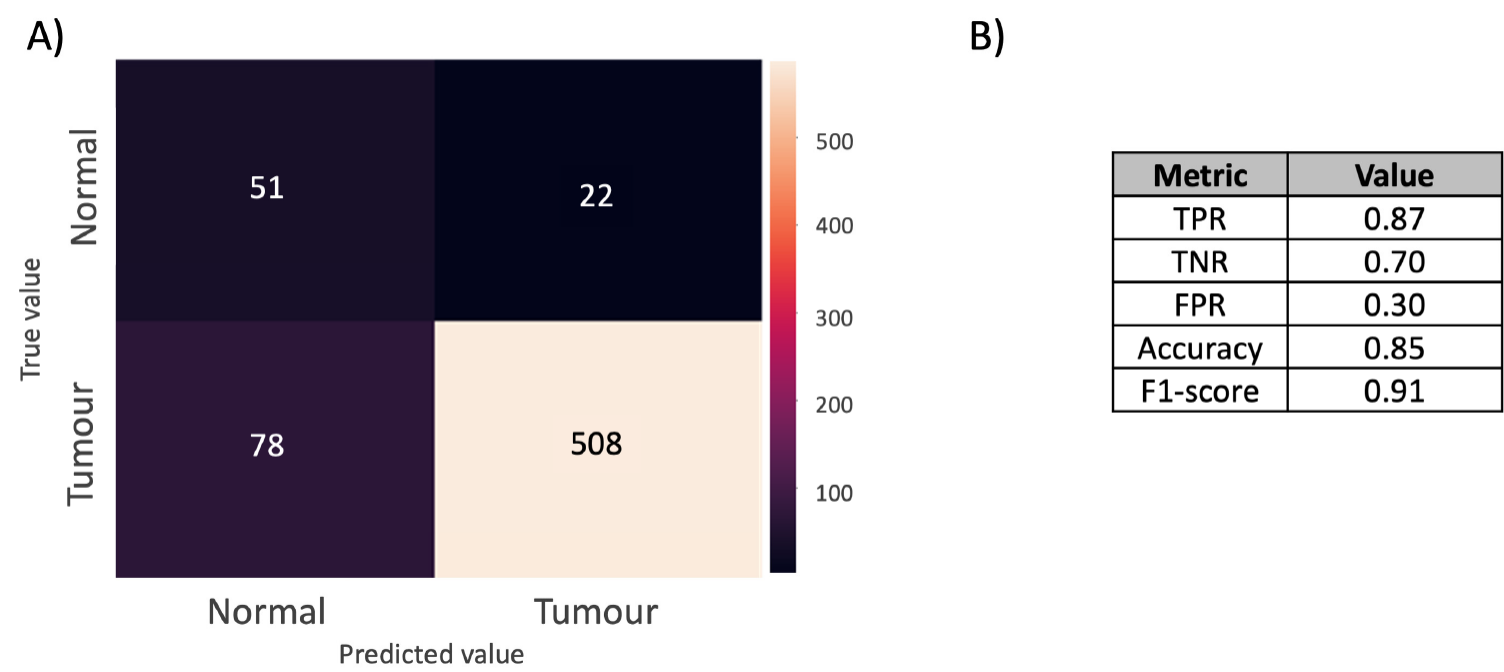
(**A**) Confusion matrix obtained from optical imaging-based DL. A core was classified as tumourous if at least 300 pixels had a probability >0.5 for class positive. The confusion matrix reports the number of true and false positives and negatives. (**B**) Model performance. The table reports the true positive rate (TPR), true negative rate (TNR), false positive rate (FPR), accuracy, and F1-score.

The resultant DL confusion matrix is presented in Figure 2A. The true positive rate, false positive rate, true negative rate, accuracy, and F1-score are reported in Figure 2B. The full results are reported in Table S2, where the metrics introduced in Section 3 are reported after considering thresholds for classifying a core as tumourous in the range between 0 and 5000 pixels. The accuracy and F1-score are, respectively, 0.85 and 0.91. The F1-score is a more appropriate metric to evaluate model performance due to the class imbalance. The true positive and negative rates were, respectively, 0.87 and 0.70, which shows that the model correctly classifies both tumour and normal cores and, at the same time, that it performs slightly better at predicting positive rather than negative cores.

**Figure 3:**
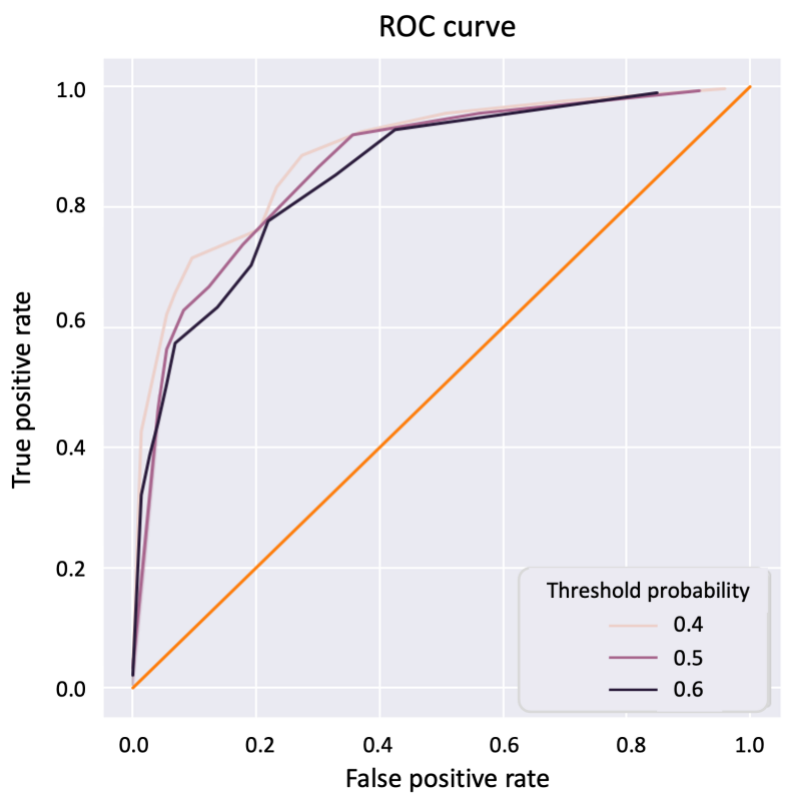
ROC-AUC curve to illustrate the deep learning model performance. An ROC-AUC curve was obtained by using a range of thresholds (0.4–0.6) on the positive probabilities and by using all thresholds between 0 and 5000 on the number of pixels necessary to classify a core as tumourous.

A receiver operating curve - area under curve (ROC-AUC) analysis was performed by placing a threshold of 0.5, 0.4, or 0.6 on the probabilities of a positive prediction, and individual data points are generated by varying the criterion between 0 and 5000 with the number of pixels detected as positive that are necessary to classify a core as tumourous. The ROC curve is presented in Figure 3 with a corresponding AUC of 0.87, 0.85, and 0.70 for thresholds of, respectively, 0.5, 0.4, and 0.6.

While the DL model has demonstrated a robust and high performance when validated with the completely independent FFPE TMA data, it should be noted that it also suffers from some underlying limitations. Firstly, the performance can still be improved, for instance, by including TMA data for training. While the addition of TMA cores in the training dataset could straightforwardly improve the model’s performance, using exclusively TMA cores in the training, however, might be difficult to achieve due to the high number of samples and tiles required to be able to train a DL algorithm that performs well. Indeed, in order to achieve a highly accurate model, it is believed that around 10,000 samples are required [11], and only 1032 images were available for this study. Apart from the obvious requirement of data in high quality and quantity, this represents a more general challenge in terms of concept drift [28] and indicates that a large amount of information (in this case, morphological information) is needed for the DL model to ‘understand’ the predictive problem, thus leaving space for improvement in terms of the specificity of the information obtained. In addition, despite the ability of DL models to capture more complex patterns when adequately trained, they do require more computational resources compared to shallow learning models, and their classification mechanisms are not easily interpretable.

### 2.2. DESI-MSI-Based Shallow Learning Approach

**Figure 4:**
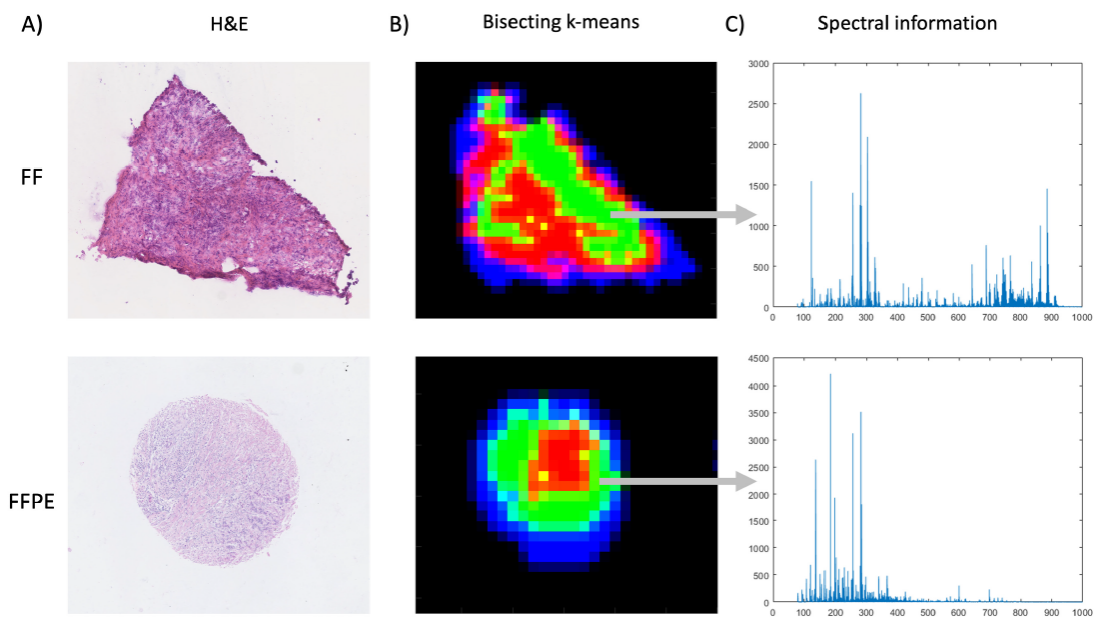
Spectral information comparison in FF and FFPE breast cancer tissue samples. (**A**) Corresponding H&E images of the FF and FFPE breast cancer tissue samples being compared. (**B**) The K-means image segmentation approach was used to visualise similar regions from the raw hyperspectral datasets by assigning a false colour to each identified spectral cluster. Specifically, the green clusters (in both cases) are tumour regions, red clusters are tumour stroma, blue clusters are the tissue background, and black clusters are the slide background. (**C**) Spectral information (50–1000 *m*/*z*) illustrated by the mean spectra extracted from breast tumour regions (green) of FF and FFPE tissue samples. The spectral intensities are raw unnormalised counts.

Before comparing the classification performance, it is worth examining the additional dimension of information that MSI provides in the spectral domain. Traditionally, MSI has been most commonly applied on fresh frozen (FF) tissues, as the use of FFPE samples for metabolic research was anticipated to be challenging due to some of the molecular content being lost because of the amount of ethanol gradient to remove water during sample preparation. To evaluate the extent of this effect and hence its impact on predictive modelling, the spectral characteristics of the FFPE MSI dataset used for subsequent classification were first compared to those of a corresponding dataset obtained from comparable FF tissues. To visualise any change in spectral information compared to the more commonly used FF samples, spatio-chemical structures of the FF and FFPE datasets were extracted from similar tumourous areas by using the k-means segmentation approach [29] (Figure 4). After pre-processing, 908 *m*/*z* and 158 *m*/*z* values were detected in the FF and FFPE samples, respectively, where 26% (41 of 158) of the peaks between FF and FFPE were found to be shared within a tolerance of 10 ppm. By inspection of their respective centroid spectra that correspond to a tumourous region, the FFPE case shows an intensity reduction of features, especially for (phospho)lipids (600–900 *m*/*z*), which agrees with previous findings stating that processing FFPE samples with various solvents removes metabolites [5,30]. While not as strong as in FF samples, FFPE samples nevertheless exhibit a fair amount of lipid signals. These results are in line with findings by Hughes et al. [31], who reported that solvent-resistant lipids remained in formalin-fixed tissue. Previous studies have reported up to 72% overlap of metabolites in FF and FFPE samples, but in those cases, analytical platform-matrix-assisted laser desorption/ionisation mass spectrometry imaging (MALDI-MSI) was used [5,32,33]. The ionisation process of MALDI-MSI and DESI-MSI is intrinsically different, and the former involves the use of a matrix, which leads to the formation of matrix ion artefacts, which may pose an additional challenge in FF samples. Specifically, ions from low-molecular-weight metabolites (50–400 *m*/*z*) can be suppressed by the abundance of fatty acids and complex lipids, which in the case of MALDI becomes negligible as their signal is overwhelmed by the matrix and matrix fragment peaks. On the other hand, when lipid signals are significantly reduced because of the ethanol gradient during the sample processing of FFPE samples, high levels of low-molecular-weight molecules as well as fatty acids become more prominent in the mass spectrum [34,35,36].

**Figure 5:**
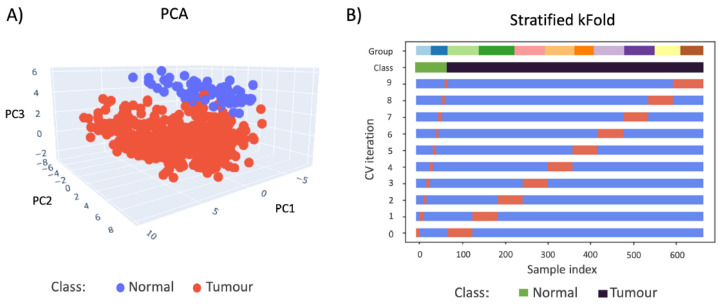
(**A**) 3D PCA plot (first 3 components) visualisation of normal (blue) and cancer (red) FFPE core MSI data. PC1 = 34.60%, PC2 = 25.97%, and PC3 = 7.90% (**B**) The cross-validation behaviour is visualised and colour-coded to display the imbalance in distribution. The group colour codes show the number of samples in each TMA. The data was split 10 times, and the samples chosen for training (blue) and testing (orange) are clearly indicated for each iteration of CV.

Owing to the plentiful information obtainable from these fatty acids, as well as from the select lipid species that are stable in FFPE samples [37], it is reasonable to assume that the spatial mapping of all these biochemical features using DESI-MSI may provide greater, more specific diagnostic power than the optical modality alone. Indeed, a clear linear separation is observed when the MSI data obtained from FFPE breast TMAs are visualised by principal component analysis (PCA) (Figure 5A). The PCA score plot reveals that the spectral characteristics of normal and tumourous tissue cores are clearly distinguishable from each other, which is almost exclusively shown by the third principal component (7.90% of total variance, with 34.60% for PC1 and 25.97% for PC2, respectively). To demonstrate the statistical significance of this observed separation, a Mann-Whitney test was conducted on this principal component, which produced test statistics of U=339 and p<0.05 (two-tailed). As some material is always consumed during MSI, the thinly cut FFPE sections (4 µm) in this case could not be used for further staining due to the large number of missing and incomplete cores after DESI-MSI. Similarly, due to the limited number of sections available, it was not possible to generate another balanced, independent test set to evaluate the robustness of the trained model as in the deep learning case. As a result, an LR model trained on these data was evaluated by cross validation only. Figure 5B illustrates the imbalanced distribution of the sample classes, as well as the cross-validation behaviour over 10 iterations of the LR model training. It can be seen that data from multiple slides are always used in training and testing, which is essential in avoiding the bias introduced due to the intrinsic unfair distribution of cancer and normal cores on slides.

**Figure 6:**
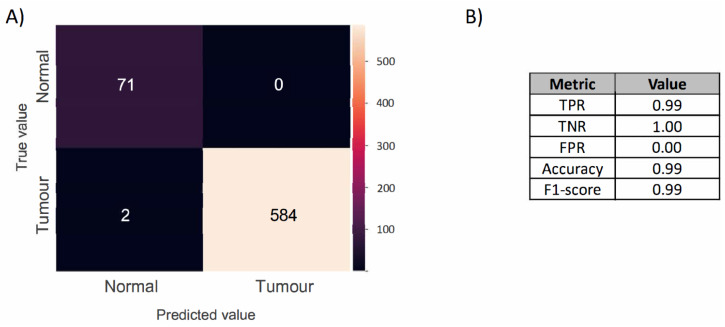
(**A**) Confusion matrix of the MSI-based LR classification model produced by the cross-validating normal and cancerous FFPE samples, comparing the predicted label (*x*-axis) with the true label (*y*-axis), with true positives appearing along the matrix diagonal. (**B**) Model performance. The table reports the true positive rate (TPR), true negative rate (TNR), false positive rate (FPR), accuracy, and F1-score.

Figure 6 shows that a higher classification performance was achieved by the resulting model when compared to the optical data-based model, misclassifying only two cancerous cores, with a balanced accuracy of 0.99 and an F1-score of 0.99 (TPR = 0.99, TNR = 1.00, FPR = 0), albeit based on cross validation alone.

Additionally, this FFPE cohort includes relatively old samples from as early as 1935, and the newest samples were collected in 2013. Sample age is another consideration that is frequently raised in clinical work; however, our previous study on the metabolic effect of sample age [37] indeed showed that the intensity of metabolites in the lower mass range (100–500 *m*/*z*) decreases with age, while metabolites in the higher mass range (500–900 *m*/*z*) remain relatively stable over time (Figure S1). Despite a decreased signal in the lower mass range, it seems not to have an impact on classification of the FFPE samples, highlighting that FFPE samples have sufficient biochemical information for not only diagnostic but also biomarker and therapeutic discoveries. As FFPE samples have been stored for decades in institutes all over the world, these results could further expand the study of rare diseases where sample availability is limited and samples are available in FFPE archives.

Further investigation was therefore carried out to determine the features involved in classification. Important features were extracted from the dataset using LR coefficients generated by the classification model for each feature and univariate analysis of variance. A total of 48 features were found to be significant in predicting the FFPE breast TMA samples, and some have been tentatively identified via literature search and reversed-phase liquid chromatography mass spectrometry in the case of lipids (Table S3). Amongst these, there are several fatty acid species, which have previously been reported to have increased signals in breast tumour tissue compared to normal breast tissue [38]. We also note the emergence of one specific lipid species, LPI(18:0) (599.32 *m*/*z*), which coincides with a previous study [23] that also reported its increased expression in breast tumour tissue using DESI-MSI.

**Figure 7:**
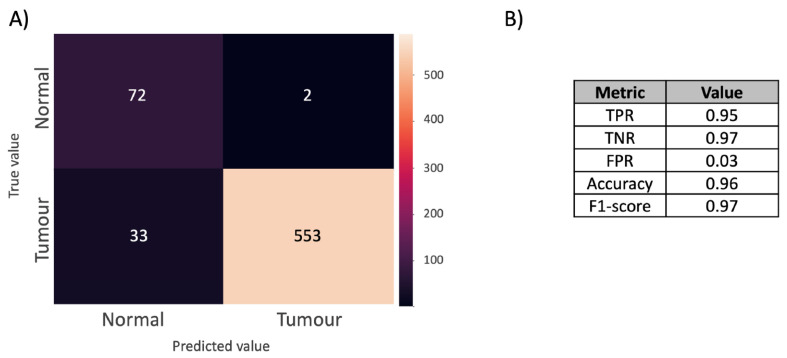
(**A**) Confusion matrix of the final MSI LR classification model based on only significant features. The model is produced by the cross validation of normal and tumour breast cores, comparing the predictive label (*x*-axis) against the true label (*y*-axis). (**B**) Model performance. The table reports the true positive rate (TPR), true negative rate (TNR), false positive rate (FPR), accuracy, and F1-score.

**Figure 8:**
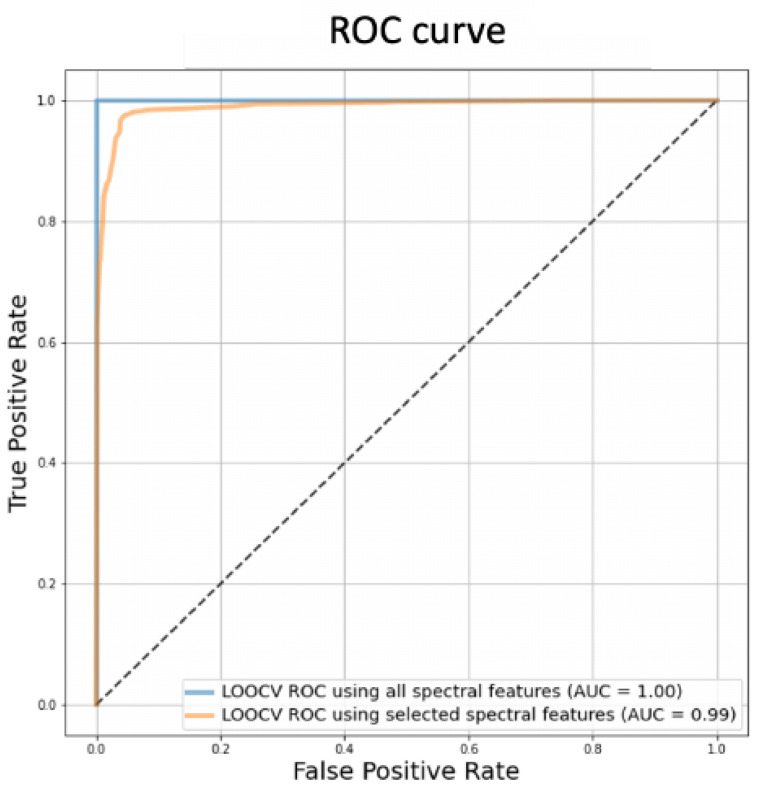
ROC-AUC curves for MSI-based LR models before (blue line) and after feature selection (orange line).

With further validation, these features can thus be considered to be potential biomarkers for the automated diagnosis of FFPE TMA samples. By generating a new LR model using only these significant features, the new confusion matrix (Figure 7) shows the performance of the model, where a balanced accuracy of 0.96 and an F1-score of 0.97 (TPR = 0.95, TNR = 0.97, FPR = 0.03) were achieved. ROC curve analysis was applied on the LR models before and after feature selection (Figure 8), which shows that feature selection retains the model performance with AUC reducing from 1.0 to 0.99 (Figure 8), suggesting that a robust model free of over-fitting is obtainable.

Naturally, the MSI-based approach also has its limitations. Compared to the optical imaging-based gold standard, which can resolve sub-micron features, the pixel size of 85 µm (and hence the spatial resolution) used in this study is orders of magnitude inferior. Although this was evidently sufficient for identifying the existence of malignancy, the identification of isolated tumour cells (about 20 µm in diameter) is not feasible at this level of resolution, making comparison with IHC images difficult. While a resolution as high as 20 µm has been described in the literature for DESI-MSI [39], this resolution mismatch does present a challenge for the interpretation of the data in conjunction with the current gold standard. In this vein, MALDI and secondary ion mass spectrometry have been reported to provide appropriate resolution for single cell identification; however, the higher spatial resolution also increases the analysis time, making the method impractical for clinical applications.

Despite the lower resolution, the outlined DESI-MSI approach is reasonably quick when compared to the optical workflow. In principle, a 1 cm^2^ tissue section can be analysed in 5–10 min on a commercially available Time-of-Flight mass spectrometer. In comparison, the optical scanning of a similar area would take 35–60 min, depending on the scanning mechanism used [8]. The analysis speed can be further improved by using more sensitive instrumentation and restricting the investigation to a well-defined panel of metabolic markers.

## 3. Materials and Methods

### 3.1. Materials

A total of 11 FFPE TMA blocks were obtained from the Department of Pathology at Landspitali, the National University Hospital in Iceland (Reykjavik, Iceland). After initial assessment by a trained histopathologist, annotations were assigned to cores that contained sufficient pathologically relevant tissue types. Out of these, nine blocks included 586 breast cancer tissue cores from 214 patients (1–6 cores per patient) [40], while two other blocks included 73 adjacent normal breast tissue cores from 27 individuals (3–4 cores per individual). All 11 TMA blocks used for imaging included a kidney and a liver core that were used as controls, and the kidney cores were used to scale the data intensity (see Section 3.3.1). Additionally, 44 FF breast tumour and normal samples were obtained from the same department. The samples were hydrogel-embedded [41] into 7 TMAs including 2–5 sections of either breast cancer tissue or adjacent normal tissue randomly distributed in each block. The FFPE samples were sectioned at 4 µm by the hospital, and the FF TMA blocks were cryosectioned to 12 µm and stored at −80 °C until use. The study was approved by the Icelandic Bioethics Committee (reference number: VSNb2017030012-03.03).

### 3.2. Deep Learning

#### 3.2.1. Optical Imaging

To generate the test data for DL, consecutive slides obtained from the same FFPE TMA block used for MSI were stained and scanned with a digital slide scanner (NanoZoomer2.0-HT, Hamamatsu City, Japan). A high-resolution objective (40×) was used for all (*n* = 11) slides. After an initial autofocus procedure to identify the optimal focal positions of the cores, each slide was imaged within 10–20 min depending on the size of the effective ROI.

#### 3.2.2. Training Data

The algorithm was trained on 1032 whole-slide images (WSIs), which were part of three different datasets with its corresponding annotations: (1) 270 images from CAMELYON16 [42], (2) 475 from CAMELYON17 [43], and (3) 287 from in-site produced data. WSIs in this training set were pre-processed according to the steps outlined in Giunchiglia et al. [44], which resulted in 8,899,519 tiles, each measuring 224 × 24 pixels. The following algorithms were applied to the training data: (1) background homogenisation, (2) the detection of blue dye and ink, (3) the detection of green dye and ink, (4) the detection of yellow dye and ink, (5) the detection of bubbles, (6) the detection of tissue fold, (7) the detection of grey ink, coverslip edge, and broken glass, (8) the detection of black regions, (9) the detection of red dye and pen ink, (10) tissue and non-tissue segmentation, (11) the detection of out-of-focus images, (12) tiling and tile selection, and (13) stain normalisation. The DL algorithm was then tested on 11 FFPE TMA H&E slide images produced on-site, which served as an independent test set, and, prior to pre-processing, the slides were split into smaller patches, where each patch would contain one single core, to be processed separately. The splitting into smaller patches was realised first by an automated approach and by further manual curation. Additional manual quality control was completed to ensure that the correct set of cores were included in the analysis. As there were fewer artefacts in these 11 TMA images compared to the training set images, only background homogenisation, tissue and non-tissue segmentation, and tiling and tile selection were performed. Only tiles with less than 60% background were kept.

#### 3.2.3. Algorithm

The deep learning algorithm was first implemented by Campanella et al. [11] but was characterised by two modifications, namely, it runs in parallel across multiple GPUs and does not require the use of OpenSlide to access the slides, since the input consists of pre-processed tissue tiles saved as hierarchical data format version 5 (HDF5) files. The algorithm consists of a convolutional neural network (CNN) based on a multiple instance learning (MIL) approach, which corresponds to a weakly supervised method. MIL defines a set of slides $S_{i}$, with *i* = 1, 2 *…n*, as either tumourous or normal. Each $S_{i}$ is characterised by *n* instances $I_{i}$, which corresponds to tiles. If the slide $S_{i}$ is labelled as tumourous (positive), then at least one of the instances $I_{i}$ is tumourous. Instead, if $S_{i}$ is annotated as normal (negative), then none of the instances $I_{i}$ is tumourous. This approach is necessary due to the lack of comprehensive annotation at the tile level. The CNN architecture was based on Resnet24, and the model was initialised with the weights trained on ImageNet. In the inference step of the MIL training, the probability of class positive is determined for each tile. For each slide, the tile with the highest positive probability is extracted, and these *n* tiles, with an *n* equal to the number of slides, are compared to the slide level annotation in order to compute the cross entropy loss. A weighted cross entropy loss was used to correct for class imbalance, and empirical weights of 0.6 and 0.4 were set, respectively, for class positive and negative, based on the numbers of positive and negative samples. The learning rate was 0.001, the loss was minimised through a stochastic gradient descent, the model was trained for 9 epochs, and the Adam optimiser was used. In total, the algorithm required 8 GPUs, and 124 GB of memory to be trained and was implemented in PyTorch [45]. The output of the algorithm is a prediction at the tile level, where a prediction threshold was set such that one entire core was classified as tumourous if at least 300 pixels were positive within the tile and vice versa.

#### 3.2.4. Model Performance Evaluation

The model performance was evaluated throughout by computing the standard metrics, including the true positive rate (TPR), true negative rate (TNR), false positive rate (FPR), balanced accuracy, and F1-score, and through ROC-AUC curves. Once the probability of class positive for each tile was predicted, a heatmap showing the probability that the original TMA slide was class positive was reconstructed, by using the grid coordinate information of the tiles. Each tile was represented as a 224 × 224 region within the image, since 224 × 224 was the original size of the extracted tiles. The heatmap was scaled to a thumbnail, with a size fixed at 4000 pixels, and then scaled according to the aspect ratio of the original image. The heatmap was binarised using a threshold of 0.5, and only the regions within the reconstructed image where the probability of class positive was greater than 0.5 were marked as tumourous.

### 3.3. Shallow learning

#### 3.3.1. DESI-MSI Analysis

DESI-MSI analysis was performed on 4 µm sections of FFPE and 12 µm FF TMAs using a XEVO G2-XS Qtof mass spectrometer (Waters, Milford, MA, USA) controlled by MassLynx software (Waters, Milford, MA, USA). The mass spectrometer was coupled to a two-dimensional DESI stage from Prosolia Inc. (Indianapolis, IN, USA) and set at a pixel size of 85 µm. The NanoAcquity binary solvent manager (Waters Corporation, Milford, MA, USA) was used to deliver the solvent, 95:5 MeOH (Sigma-Aldrich, St. Lewis, MO, USA):H_2_O (Thermo Fisher Scientific Inc., Waltham, MA, USA) + 0.0001% raffinose, to the electrospray at a flow rate of 1.5 µL/min. Detailed information about the MS instrumental parameters can be found in Table S1. Prior to the DESI-MSI analysis, FFPE TMAs were deparaffinised by incubating the samples for 1 h at 60 °C, rinsing with xylene (2 × 8 min) and air-drying in a fume-hood overnight as described by Ly et al. [33]. Due to the deterioration of the DESI-MSI analysed tissue, consecutive FFPE and FF TMAs were stained with H&E for optical imaging.

#### 3.3.2. MSI Data Pre-Processing

An in-house Python pipeline was used first to pre-process raw data. In concise form, the pipeline consists of (1) a signal-to-noise ratio (SNR)-based peak detection procedure, (2) region-of-interest (ROI) selection via segmentation, and (3) peak alignment and recalibration. As a result, the intra-data (between pixels) and inter-data (between MS runs) variabilities were removed. Only features from tissue-specific regions were kept to reduce the effective data size. Features that were characterised as noisy or unlikely according to spatial distributions were filtered by means of the R package SPUTNIK [46]. Finally, a single data matrix of dimension M × N was produced as output for each run, where M is the total number of pixels, and N is the length of the common mass axis, which was shared between data from all runs.

To further reduce the possible batch effect, the reference cores on each slide (i.e., the kidney) were used to perform intensity scaling, and the resulting data also underwent median fold change scaling to stabilise the variance [47]. To enable the subsequent supervised analysis, a clinical pathologist manually annotated cancerous and normal tissue cores on the accompanying H&E stained optical images with clinicopathological information that allowed for more in-depth data mining. To correlate the optical and chemical images, total-ion-count images from each slide were co-registered with their corresponding H&E images, specifically by the use of affine transformation by gradient descent [27]. Average spectra per core were assigned labels accordingly and used for predictive modelling.

#### 3.3.3. Supervised Shallow Learning & Statistical Analysis

Due to the clear imbalance in the number of samples for cancer and normal tissues, a cost-sensitive approach [48] was used to weight each group accordingly during model training. As such, a weighted logistic regression (LR) classification model was built using the pre-processed MSI data, and its performance was assessed using stratified K-Fold (K = 10) cross validation to predict a core as either cancerous or normal [49]. The cross-validation training and testing sets were chosen such that the slide-to-slide bias was minimised. The model performance was evaluated by the same metrics used for deep learning for easy comparison. In addition, model refinement was carried out by performing the log likelihood ratio test to reject the null hypothesis that a given spectral feature was not significant in the classification of the data using LR, for all spectral features. To visualise comparison between models, the receiver operator characteristic (ROC) curve using different probability thresholds in the LR model was plotted, hence; its corresponding area under the curve (AUC) was used as the metric.

The labelled data matrices were also analysed univariately, in the form of a Kruskal–Wallis test. A threshold of (*p* < 0.05) was used to select significantly different features in the intensity domain, which was followed by false discovery rate correction with the Benjamini–Hochberg procedure. Both the multivariate and univariate features were then compared. Overlapping features from the two approaches were ultimately considered features of interest and used in constructing the optimised model. Due to a limited amount of clinical tissues, the use of well-established online databases [50,51,52] as well as previous publications were used for only tentative metabolic annotations.

## 4. Conclusions

With visual analysis of H&E-stained histological sections using a traditional microscope being the cornerstone of pathological diagnostics for the past century, there is a need for more rapid, automatic, and reliable diagnostic methods. DL-assisted analysis of histological optical images has led the way in recent years towards a truly automated workflow, as we demonstrate here that a weakly supervised deep learning method can be used to provide diagnoses of breast cancer FFPE TMA samples with an overall F1-score of 91%. For this approach to be routinely used for clinical studies, however, a large amount of high-quality training data is still needed due to the lower specificity in the image contrast. Alternatively, the chemically specific contrast from MSI provides a cross-validated predictive accuracy of close to 100% based on the F1-score obtained from shallow learning approaches that are easy to interpret. While not directly comparable to the DL results, model optimisation via feature refinement has revealed chemical species that are correlated with the underlying biology, which can potentially be used as biomarkers to build a robust model that can be used across data obtained from different sample types and experiments, once validated. Thanks to the $10^{2}$–$10^{3}$ channels that are available from hyperspectral imaging of this kind, this even unveils the possibility of more in-depth data mining based on the associated pathological information of the patients, potentially shedding light on the pathways and networks that drive different strata of cancer, e.g., subtypes, age, grade, etc. Finally, it should be noted that the two approaches presented here are not mutually exclusive. In fact, future research may well make use of the accessibility of FFPE samples to enable DL approaches based on MSI data. Likewise, optical images as an established standard could also be used jointly with MSI in building more accurate predictive models based on the chemical information, with the use of novel approaches such as manifold alignment for knowledge transfer [53].

## Data Availability

The data that support the results of this study are not publicly available due to ethical reasons, but are accessible from the corresponding authors upon reasonable request. The code for the pre-processing of FFPE TMA images is available at (https://github.com/valegiunchiglia/tma, accessed on 19 April 2022).

## Supplementary Materials

The following supporting information can be downloaded at https://www.mdpi.com/article/10.3390/metabo12050455/s1, refs. [37,54,55,56] are cited in the supplementary materials. Table S1: Instrumental parameters for DESI-MSI; Table S2: Model performance. In order to classify a core as tumour, different thresholds on the minimum number of positive pixels within the core were used. Only the core with a positive pixel number above the chosen threshold were classified as tumour. The table reports the number of true positive (tp), true negative (tn), false positive (fp), false negative (fn), together with the true positive rate (TPR), false positive rate (FPR), true negative rate (TNR), accuracy (ACC) and F1-Score (F1); Table S3: Features of interest in FFPE breast TMAs, Figure S1: The effect of sample age on the metabolic information extracted from formalin-fixed and paraffin embedded tissue sample.

## Abbreviations

The following abbreviations are used in this manuscript:

AUC	Area under the curve
CAD	Computer-assisted diagnostics
CNN	Convolutional neural network
DESI	Desorption electrospray ionisation
DL	Deep learning
FF	Fresh frozen
FFPE	Formalin-fixed and paraffin-embedded
FPR	False positive rate
HDF5	Hierarchical data format version 5
H&E	Haematoxylin and eosin
IHC	Immunohistochemistry
LR	Logistic regression
MALDI	Matrix-assisted desorption/ionisation
MIL	Multiple instance learning
MS	Mass spectrometry
MSI	Mass spectrometry imaging
PCA	Principal component analysis
ROC	Receiver operating characteristic
ROI	Region-of-interest
SNR	Signal-to-noise ratio
TNR	True negative rate
TMA	Tissue microarray
TPR	True positive rate
WSI	Whole-slide image
