# Supplementary material for: Automated Cancer Diagnostics via Analysis of Optical and Chemical Images by Deep and Shallow Learning
Source: Metabolites. 2022 May 18;12(5):455. doi: 10.3390/metabo12050455 (PMC9143055; doi:10.3390/metabo12050455)
Supplement: Supplementary file 1 [file metabolites-12-00455-s001.zip › metabolites-1713773-supplementary.pdf]

Supplementary:

Supplementary Table S1: Instrumental parameters for DESI-MSI

**Parameters**	**Xevo G2-XS QTOF (Waters Corporation, Milford, MA, USA)**
Scan time	1 second
Scan mode	Sensitivity
Mass analyser	TOF
Mass range	50 – 1000 m/z
Solvent flow rate	1.5 µl/min
Spray voltage	4.5 kV
Gas inlet pressure	7 bar
Sprayer-to-surface distance	2 mm
Sprayer-to-inlet distance	10 mm
Sprayer incidence angle	75°
Pixel size	85 μm^2
Source temperature	120 °C
Ion mode	Negative
Sampling cone voltage	-40 V
Source offset	-80 V

Supplementary Table S2: ***Model performance.*** *In order to classify a core as tumour, different thresholds on the minimum number of positive pixels within the core were used. Only the core with a positive pixel number above the chosen threshold were classified as tumour. The table reports the number of true positive (tp), true negative (tn), false positive (fp), false negative (fn), together with the true positive rate (TPR), false positive rate (FPR), true negative rate (TNR), accuracy (ACC) and F1-Score (F1).*

Threshold area	tp	tn	fp	fn	TPR	FPR	TNR	ACC	F1
0	551	7	66	3	0.99	0.9	0.1	0.89	0.94
100	528	37	36	26	0.95	0.49	0.51	0.9	0.94
200	506	47	26	48	0.91	0.36	0.64	0.88	0.93
300	472	54	19	82	0.85	0.26	0.74	0.84	0.9
400	433	58	15	121	0.78	0.21	0.79	0.78	0.86
500	400	62	11	154	0.72	0.15	0.85	0.74	0.83
600	369	68	5	185	0.67	0.07	0.93	0.7	0.8
700	337	69	4	217	0.61	0.05	0.95	0.65	0.75
800	302	71	2	252	0.55	0.03	0.97	0.59	0.7
900	264	71	2	290	0.48	0.03	0.97	0.53	0.64
1000	232	72	1	322	0.42	0.01	0.99	0.48	0.59
1200	133	73	0	421	0.24	0.0	1.0	0.33	0.39
1400	70	73	0	484	0.13	0.0	1.0	0.23	0.22
1600	28	73	0	526	0.05	0.0	1.0	0.16	0.1
1800	14	73	0	540	0.03	0.0	1.0	0.14	0.05
2000	7	73	0	547	0.01	0.0	1.0	0.13	0.02
2200	2	73	0	552	0.0	0.0	1.0	0.12	0.01
2400	2	73	0	552	0.0	0.0	1.0	0.12	0.01
2600	0	73	0	554	0.0	0.0	1.0	0.12	0.0
2800	0	73	0	554	0.0	0.0	1.0	0.12	0.0
3000	0	73	0	554	0.0	0.0	1.0	0.12	0.0
3200	0	73	0	554	0.0	0.0	1.0	0.12	0.0
3400	0	73	0	554	0.0	0.0	1.0	0.12	0.0
3600	0	73	0	554	0.0	0.0	1.0	0.12	0.0
3800	0	73	0	554	0.0	0.0	1.0	0.12	0.0
4000	0	73	0	554	0.0	0.0	1.0	0.12	0.0
4200	0	73	0	554	0.0	0.0	1.0	0.12	0.0
4400	0	73	0	554	0.0	0.0	1.0	0.12	0.0
4600	0	73	0	554	0.0	0.0	1.0	0.12	0.0
4800	0	73	0	554	0.0	0.0	1.0	0.12	0.0
5000	0	73	0	554	0.0	0.0	1.0	0.12	0.0

Supplementary Table S3: Features of interest in FFPE breast TMAs.[1]

Experimental m/z	Theoretical m/z	Ppm	Adduct	Chemical formula	Tentative annotations
134.0467	134.0472	3.8	M-H-	C5H5N5	Adenine
135.0452	135.0452	-0.2	M-H-	C8H8O2	Phenylacetic acid
152.0028	152.0023	-3.4	M-H-	C3H7NO4S	3-Sulfinoalanine
157.0870	157.087	-0.6	M-H-	C8H14O3	3-Oxooctanoic acid
166.0150	166.0146	-2.2	M-H-	C7H5NO4	4-Nitrobenzoic acid
177.0915	177.0921	3.6	M-H-	C11H14O2	5-Phenylvaleric acid
183.1384	183.1391	3.7	M-H-	C11H20O2	Undecylenic acid
185.1176	185.1183	4.1	M-H-	C10H18O3	10-Hydroxy-2-decenoic acid
185.1541	185.1547	3.0	M-H-	C11H22O2	Undecanoic acid
197.1182	197.1178	-1.9	M-H2O-H-	C11H20O4	Undecanedioic acid
199.1337	199.1340	1.7	M-H-	C11H20O3	9-Undecenoic acid, 11-hydroxy-, (9Z)-
211.1337	211.1334	-1.4	M-H2O-H-	C12H22O4	Dodecanedioic acid
213.1856	213.1860	1.9	M-H-	C13H26O2	Tridecanoic acid
228.2049	-	-	-	-	-
241.2171	241.2173	0.9	M-H-	C15H30O2	Pentadecanoic acid
250.1434	-	-	-	-	-
267.1974	267.1960	-5.1	M-H2O-H-	C16H30O4	Hexadecanedioic acid
269.2126	269.2122	-1.5	M-H-	C16H30O3	3-Oxohexadecanoic acid
269.2485	269.2486	0.4	M-H-	C17H34O2	Heptadecanoic acid
271.2278	271.2279	0.5	M-H-	C16H32O3	2-Hydroxyhexadecanoic acid
275.1295	275.1283	-4.5	M-H2O-H-	C16H22O5	-
283.1128	283.1131	-1.1	M-H-	C21H16O	-
299.2565	299.2592	9.1	M-H-	C18H36O3	13-Hydroxyoctadecanoic acid
301.1439	301.1445	1.0	M-H-	C18H22O4	Enterodiol
311.2225	311.2228	0.9	M-H-	C18H32O4	9,12-Octadecadienoic acid, 15,16-dihydroxy-, (*Z*,*Z*)- (9CI)
313.2381	313.2384	0.9	M-H-	C18H34O4	12-Octadecenoic acid, 9,10-dihydroxy-, (12Z)-
315.1872	-	-	-	-	-
324.2211	324.218	-9.7	M-H-	C18H31NO4	-
325.2743	325.2743	-0.1	M-H2O-H-	C20H40O4	11,12-Dihydroxyeicosanoic acid
329.2370	-	-	-	-	-
337.2360	337.2379	5.8	M-H2O-H-	C20H36O5	Prostaglandin F1a
355.1951	355.1915	-10.0	M-H-	C22H28O4	-
356.1709	356.1715	1.9	M-H-	C17H27NO7	Uplandicine
363.2154	363.2177	6.3	M-H-	C21H32O5	-
366.2264	-	-	-	-	-
373.2031	373.2020	-3.0	M-H-	C22H30O5	6α-Methylprednisolone
379.2463	379.2490	7.1	M-H-	C22H36O5	Bisnorcholic acid
385.1940	-	-	-	-	-
409.2564	-	-	-	-	-
417.2063	417.2074	-2.6	M-H-	C27H30O4	-
419.2665	-	-	-	-	-
421.3072	-	-	-	-	-
435.2552	435.2517	-7.9	M-H-	C21H41O7P	LPA(18:1)
437.1951	437.1970	4.4	M-H-	C26H30O6	-

[1] To confirm the identity of certain lipid species, specifically LPI(18:0), metabolites were extracted from FFPE tissue rolls using a 2-phase method as described in Beckonert et al. (Beckonert et al., 2007). A reversed-phase chromatography lipids method was used for LC-MS analysis as described in Lewis et al. (Lewis et al., 2022) and peakpantheR (Wolfer et al., 2021) was used to identify LPI(18:0).

485.2770	-	-	-	-	-
586.1987	586.2020	-5.6	M-H-	C32H33N3O6S	-
599.3185	599.3202	-2.9	M-H-	C27H53O12P	LPI(18:0)
692.4617	-	-	-	-	-

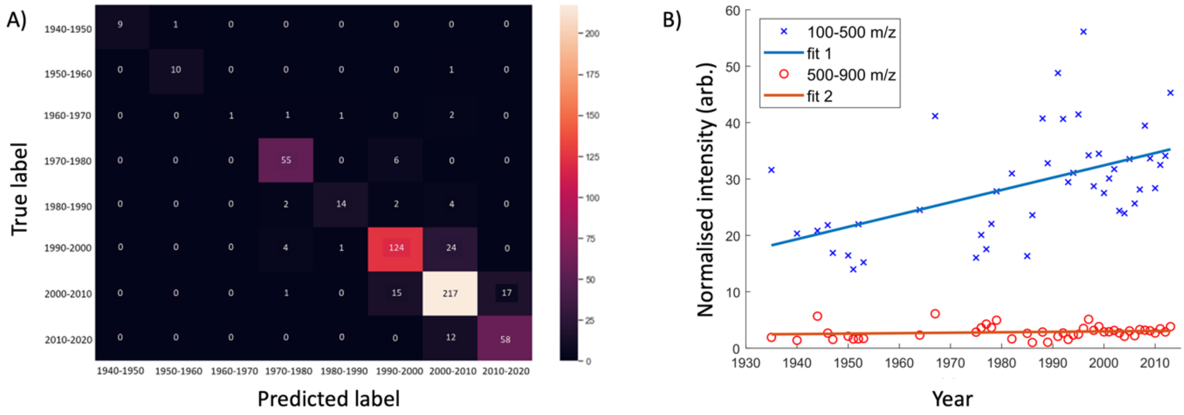

Supplementary Figure S1: The effect of sample age on the metabolic information extracted from formalin-fixed and paraffin embedded tissue sample. *A) Confusion matrix of the LR classification model produced by cross-validating the FFPE samples with 10-year interval ranging from the years 1940-2013, comparing the predicted age (x-axis) against the true age (y-axis), with true positives appearing along the matrix diagonal. B) The mean metabolic signal intensities of FFPE samples from the years 1935 - 2013. Blue crosses correspond to the lower mass range (100-500 m/z), while red dots correspond to the higher mass range (500-900 m/z). The solid lines represent the respective linear regressions performed on the data* points (Isberg et al., 2021).

References:

Beckonert, O., Keun, H. C., Ebbels, T. M. D., Bundy, J., Holmes, E., Lindon, J. C., & Nicholson, J. K. (2007). Metabolic profiling, metabolomic and metabonomic procedures for NMR spectroscopy of urine, plasma, serum and tissue extracts. *Nature Protocols*, *2*(11), 2692–2703. https://doi.org/10.1038/nprot.2007.376

Isberg, O. G., Xiang, Y., Bodvarsdottir, S. K., Jonasson, J. G., Thorsteinsdottir, M., & Takats, Z. (2021). The effect of sample age on the metabolic information extracted from formalin-fixed and paraffin embedded tissue samples using desorption electrospray ionization mass spectrometry imaging. *Journal of Mass Spectrometry and Advances in the Clinical Lab*, *22*, 50–55. https://doi.org/10.1016/j.jmsacl.2021.10.004

Lewis, M. R., Chekmeneva, E., Camuzeaux, S., Sands, C. J., Yuen, A. H. Y., David, M., Salam, A., Chappell, K., Cooper, B., Haggart, G. A., Maslen, L., Gómez-Romero, M., Horneffer-Van Der Sluis, V., Correia, G., & Takats, Z. (2022). An Open Platform for Large Scale LC-MS-Based Metabolomics. *ChemRxiv*.

Wolfer, A. M., Correia, G. D. S., Sands, C. J., Camuzeaux, S., Yuen, A. H. Y., Chekmeneva, E., Takáts, Z., Pearce, J. T. M., & Lewis, M. R. (2021). peakPantheR, an R package for large-scale targeted extraction and integration of annotated metabolic features in LC-MS profiling datasets. *Bioinformatics*. https://doi.org/10.1093/bioinformatics/btab433/6298587
